# A High-Sensitivity Low-Nanoflow LC-MS Configuration for High-Throughput Sample-Limited Proteomics

**DOI:** 10.1021/acs.analchem.3c03058

**Published:** 2023-12-13

**Authors:** Runsheng Zheng, Manuel Matzinger, Rupert L. Mayer, Alec Valenta, Xuefei Sun, Karl Mechtler

**Affiliations:** † Thermo Fisher Scientific, Dornier Str. 4, 82110 Germering, Germany; ‡ IMP—Institute of Molecular Pathology, Campus-Vienna-Biocenter 1, A-1030 Vienna, Austria; § Thermo Fisher Scientific, 1228 Titan Way, Sunnyvale, California 94085, United States; ⊥ IMBA—Institute of Molecular Biotechnology of the Austrian Academy of Sciences, Dr. Bohr Gasse 3, A-1030 Vienna, Austria; ¶ Gregor Mendel Institute of Molecular Plant Biology of the Austrian Academy of Sciences, Dr. Bohr Gasse 3, A-1030 Vienna, Austria

## Abstract

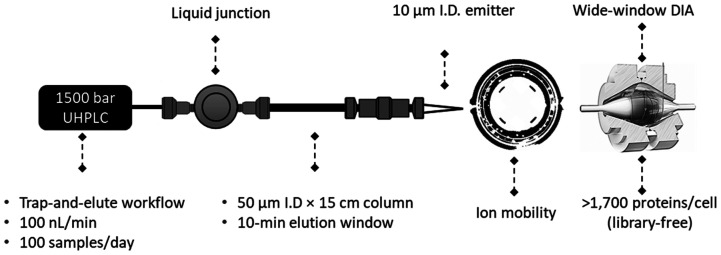

This work demonstrates the utility of high-throughput nanoLC-MS and label-free quantification (LFQ) for sample-limited bottom-up proteomics analysis, including single-cell proteomics (SCP). Conditions were optimized on a 50 μm internal diameter (I.D.) column operated at 100 nL/min in the direct injection workflow to balance method sensitivity and sample throughput from 24 to 72 samples/day. Multiple data acquisition strategies were also evaluated for proteome coverage, including data-dependent acquisition (DDA), wide-window acquisition (WWA), and wide-window data-independent acquisition (WW-DIA). Analyzing 250 pg HeLa digest with a 10-min LC gradient (72 samples/day) provided >900, >1,800, and >3,000 protein group identifications for DDA, WWA, and WW-DIA, respectively. Total method cycle time was further reduced from 20 to 14.4 min (100 samples/day) by employing a trap-and-elute workflow, enabling 70% mass spectrometer utilization. The method was applied to library-free DIA analysis of single-cell samples, yielding >1,700 protein groups identified. In conclusion, this study provides a high-sensitivity, high-throughput nanoLC-MS configuration for sample-limited proteomics.

## Introduction

Recently, proteomics has seen a significant shift toward proteome profiling of smaller and smaller sample quantities down to individual cells.^1−4^ Obtaining high-quality data from subnanogram (ng) protein samples requires exquisite sensitivity, accuracy, and precision for all stages of the proteomics workflow, from sample collection to data analysis.^5−9^ As such, state-of-the-art LC-MS technology plays a crucial role in our ability to investigate changes in the protein expression without bias. While improvements in MS instrumentation have improved data collection speed and quality, mass spectrometers are still inherently limited by the quality of the sample at the LC-MS interface. High-quality, low-flow LC gradient separations provide minimal coelution of target peptides, improving ionization efficiency (i.e., method sensitivity and, ultimately, proteome depth). Sensitivity is enhanced by utilizing ultralow LC flow rates and small inner diameter columns/emitters,^10−12^ but at the potential cost of decreasing sample throughput. Therefore, a balance between throughput and proteome depth is required for the large-scale profiling of mass-limited samples. This is achieved through optimizing LC and MS parameters for fast sample injection and loading, efficient peptide separation, ionization and precursor isolation, accumulation, and fragmentation. Ideally, most method cycle time is dedicated to MS acquisition with minimal time wasted on sample pickup and column equilibration.

To address these needs, we optimized and evaluated the performance of a low-nanoflow UHPLC separation setup and several LC-MS methods in data-dependent acquisition (DDA), wide-window-acquisition (WWA),^13,14^ and wide-window data-independent acquisition (WW-DIA) to achieve deep proteome profiling. These methods balance proteome depth, sample throughput, and MS utilization for sample-limited proteomics including single-cell proteomics analysis.

## Result and Discussion

### A High-Sensitivity and High-Throughput Configuration in Direct Injection Workflow

**Figure 1:**
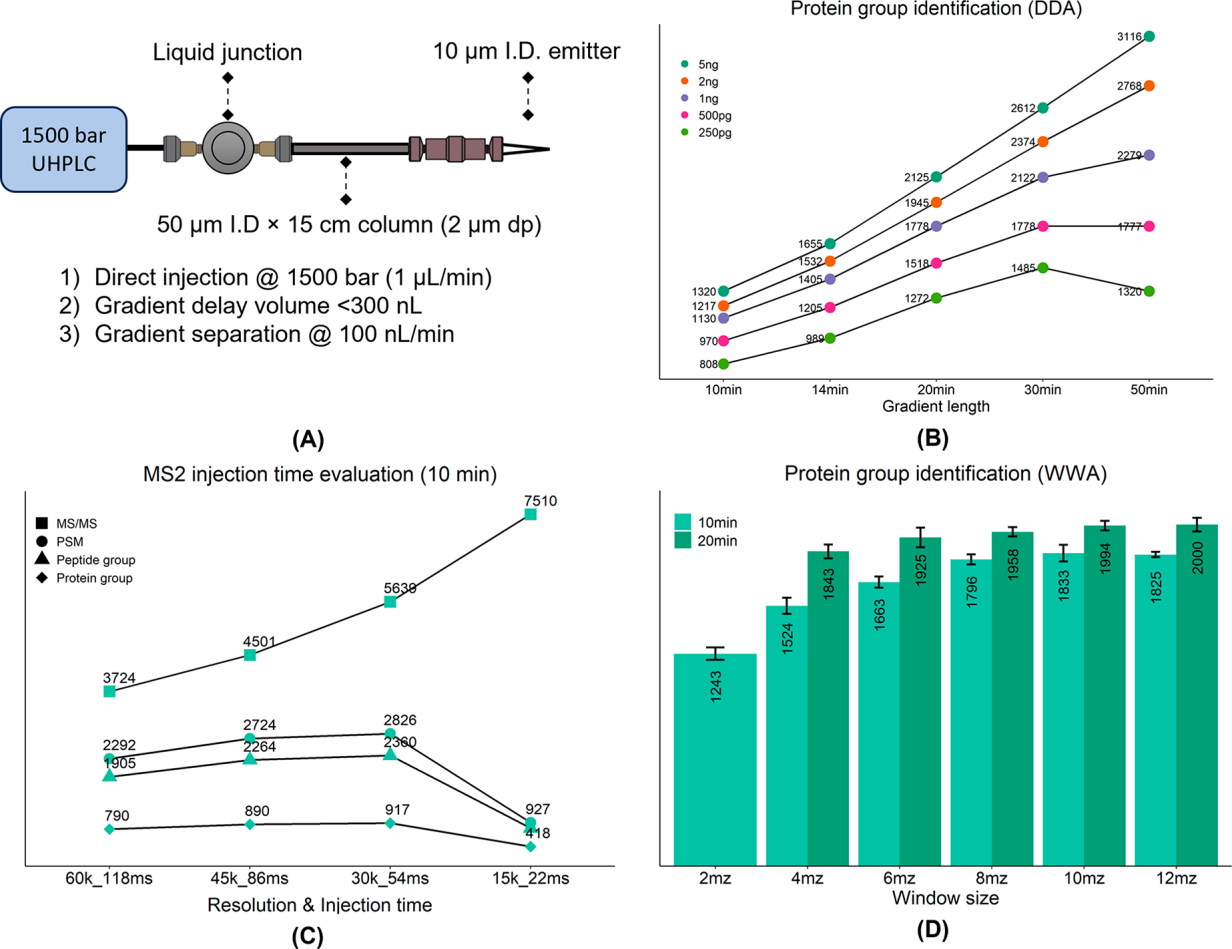
A low-nanoflow LC-MS configuration for high-performance DDA analysis in direct injection workflow. (A) Column configuration in direct injection workflow using 1500 bar for sample loading to increase sample throughput. (B) Number of protein groups identified as a function of the sample loading amount and gradient length using 118 ms IT with 60,000 resolution. (C) The fastest scan speed using 22 ms MS2 IT impacted the identifications negatively with more MS2 not being translated into PSM, peptide, and protein identifications for 250 pg HeLa digest (different experiment batch from Figure 1B). (D) 10/12 *m*/*z* isolation window significantly boosts protein group identifications using WWA approach in a 10-min gradient (72 samples/day) for 250 pg HeLa digest.

Considering multiple factors for balancing the sensitivity (flow rate and column I.D.) and sample throughput (gradient delay volume, column pressure, and volume), we established and evaluated a configuration using a 50 μm I.D. × 15 cm column in the direct injection workflow (Figure 1A). Peptide ionization is carried out via a 10 μm I.D. glass emitter into a FAIMS Pro interface operated at a single compensation voltage to reduce background ion interference. A liquid junction on the column inlet and zero-dead volume postcolumn connection to the emitter ensured stable ionization and minimal postcolumn dispersion to maintain chromatographic performance. Using this configuration, we developed five methods for fast sample loading at 1500 bar^16^ (ca. 1 μL/min at 50 °C), efficient column washing, and equilibration in 2 min while maintaining a 100 nL/min flow at a stable pressure. Each method required an additional 10 min for the sample injection cycle, column re-equilibration, and gradient delivery, enabling analysis of up to 72 samples/day (10 min gradient) (Supplemental Figure 1).

This configuration yielded <4-second peak widths (median fwhm) for the HeLa digest run using the 10-min gradient (72 samples/day), regardless of the amount of sample injected (250 pg to 5 ng), i.e., the injected amount does not exceed the column loading capacity (Supplemental Figure 2). An increasing trend in protein group identifications from 250 pg to 5 ng of HeLa digest (Figure 1B) suggests excellent method suitability for analyzing limited sample amounts in the DDA mode. We identified ∼ 1,500 protein groups from 250 pg HeLa digest in a 30-min gradient (36 samples/day) without match-between-runs. To the authors’ knowledge, this represents the most comprehensive DDA data with the highest number of identifications to date using the conventional database search algorithm.^17^ Moreover, ∼ 800 protein groups were identified using a 10 min gradient (72 samples/day), suggesting the potential for WWA and DIA strategies to further boost proteome coverage by identifying/quantifying coisolated peptides. Notably, peak broadening in the 50 min gradient (24 samples/day) reduced identifications for 250 and 500 pg samples, whereas injections with larger sample amounts (1, 2, and 5 ng) were unaffected due to more intense peaks and, consequently, more ions for sampling. Nevertheless, the low variation in protein abundance confirms reproducible LC-MS performance for protein quantification (Supplemental Figure 3), which is suitable for sample-limited proteomics, including single-cell proteomics (SCP).

### Enhanced DDA Performance by Faster MS Scan and Chimeric Spectrum Deconvolution

Given the high sensitivity achieved in this configuration, we explored the effect of MS2 scan speed on DDA performance in a 10 min gradient (72 samples/day) with a 250 pg sample (single cell level) by decreasing the injection time (IT) from 118 (parameter in Figure 1B) to 22 ms, along with the corresponding Orbitrap resolution. Encouragingly, 10–20% more peptide and protein group identifications were gained from 54 ms IT and 30,000 resolution. This results from the high-sensitivity front end (stable low-nano flow rate over the gradient, high ionization efficiency, and background ion filtering by the FAIMS Pro interface), providing sufficient ion clusters/time for faster scanning (Figure 1C). However, IT below 54 ms had the opposite impact on identifications, where more MS2 scans did not translate into more peptide spectrum matches (PSMs) and peptide and protein identifications. It illustrates that ion accumulation time and spectral resolution are crucial for precursor fragmentation and spectrum identification when analyzing limited sample amounts.

Precursor coisolation in DDA mode commonly results in complex spectra that limit the performance of conventional database search algorithms, e.g., SEQUEST, for peptide identification. By employing CHIMERYS, an advanced AI-driven algorithm for spectral deconvolution, we boosted protein identifications for 250 pg and 5 ng (10-min gradient, 72 samples/day) by 40% (1142) and 80% (2360), respectively, from the same dataset (Figure 1B and Supplemental Figure 4). However, the longer, 30 min gradient (48 samples/day) only gained 8% more protein identifications for 250 pg inputs, likely due to better chromatographic resolution between peptide peaks, yielding less complex MS spectra (Supplemental Figure 4). Further exploration with a WWA strategy in MS1 combined with CHIMERYS enabled 1,800 protein group identifications (an increase of 120%) from 250 pg HeLa digest with a 10 min gradient (72 samples/day) and 10 *m*/*z* isolation window (Figure 1D) with 118 ms MS2 injection time. This performance surpasses the previously published results in proteome depth^14^ and sample throughput,^13,14^ but also agrees with the findings of optimal window size for WWA using a completely different LC and column configuration.^13,14^

### Achieve Next-Level Performance with WW-DIA

**Figure 2:**
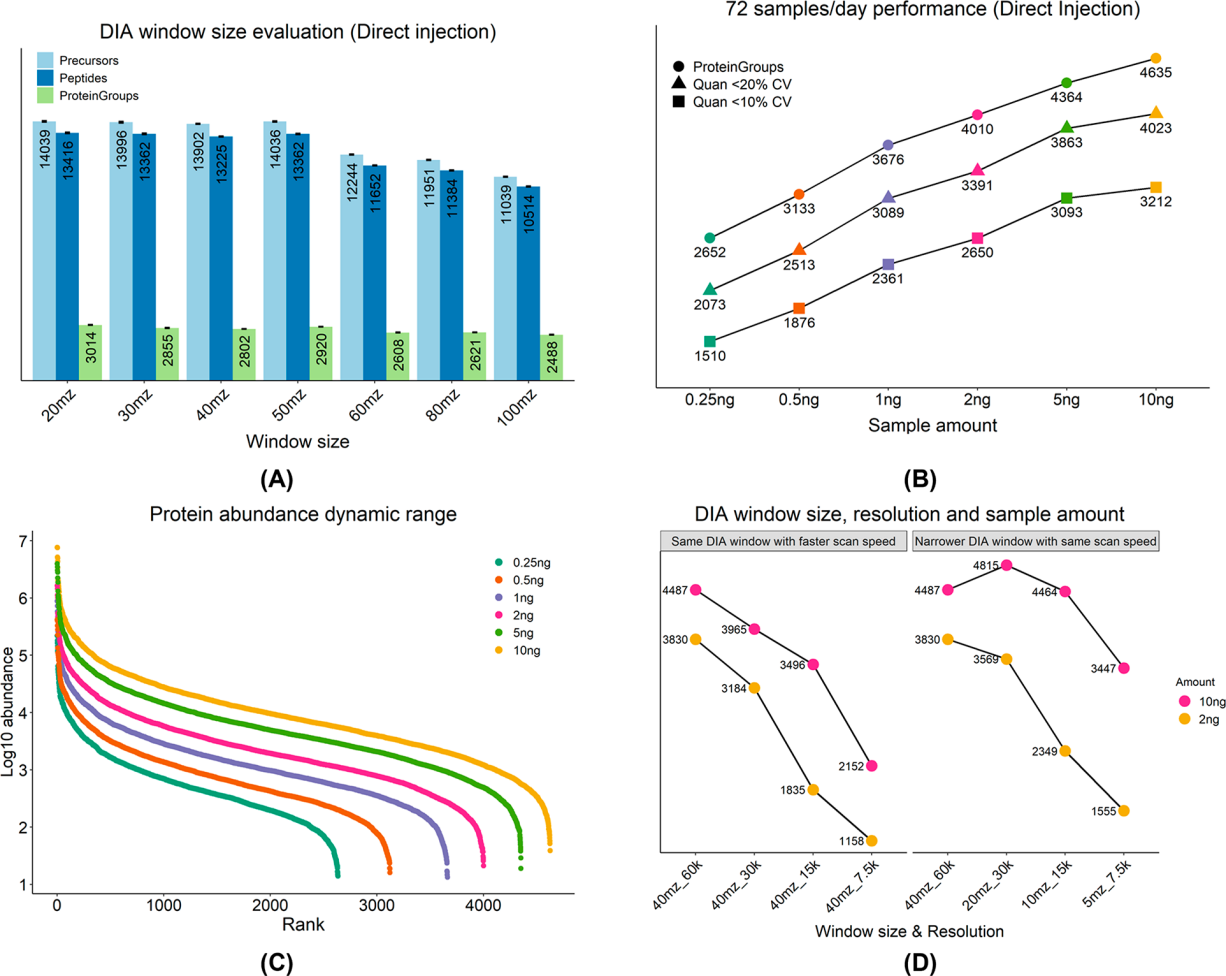
Wide-window DIA for unprecedented proteome coverage in a direct injection workflow. (A) More than 3,000 protein groups were identified from 250 pg HeLa sample using a 10-min LC gradient (72 samples/day) with 20 *m*/*z* isolation window. (B) Number of protein groups identified as a function of the sample loading amount in DIA using 40 *m*/*z* isolation window (different batch of experiment from Figure 2A). (C) The protein abundances span more than 4 orders of magnitude of the dynamic range. (D) DIA with a wider window requires a higher resolution MS2 for spectral deconvolution but still shows dependency to sample amount.

Since DIA can sample more ion population clusters for fragmentation and has seen recent advances in library-free identification algorithms, a systematic evaluation of different window sizes in DIA was performed. As many as 3,000 protein groups were identified from a 250 pg HeLa digest using a 10-min LC gradient (72 samples/day) applying a 20 *m*/*z* isolation window (Figure 2A). A decreasing identification trend illustrates the challenge of deconvoluting extraordinarily complex spectra by using wider isolation windows. Nevertheless, the WW-DIA approach enables deep proteome identification covering > 4 orders of magnitude dynamic range (Supplemental Figure 5). To illustrate the performance advantage of WW-DIA over WWA at the same MS2 scan speed, we employed the fixed 40 *m*/*z* isolation window for all the following experiments. An increasing trend in protein identification was seen with increased sample amount (250 pg to 10 ng) where > 4,600 protein groups were identified, of which 87% were quantified at < 20% coefficient of variation (CV) covering > 5 orders of magnitude of dynamic range from 10 ng sample with a 40 *m*/*z* isolation window (Figure 2B and 2C).

Due to the high sensitivity achieved from the subnanogram samples, we further explored the relationship of the MS2 IT, resolution, MS1 window size, and injection amount for larger sample quantities (Figure 2D). In short, reducing the resolution and corresponding IT (i.e., increasing scan speed) decreased protein Identifications and quantification for 2 and 10 ng samples, illustrating that the MS2 complexity with a 40 *m*/*z* isolation window requires 60,000 resolution and 118 ms IT for spectral deconvolution. While the scan speed is kept, the window size must be coordinated with the sample amount to reflect spectral complexity and ion intensity to enable optimal protein identification. For instance, a window size of 20 *m*/*z* increased protein identifications (4,815) by 10% for a 10 ng sample using an MS resolution of 30,000 in a 10 min gradient (72 samples/day). Moreover, the longer 20-min gradient (48 samples/day) does not significantly increase protein identifications (4,848 protein groups with 10 ng HeLa digest), indicating that a 10-min gradient (72 samples/day) is sufficient for WW-DIA-based sample-limited proteomics (Supplemental Figure 6).

### Increasing Sample Throughput for LFQ-DIA Profiling of Single-Cell Samples

**Figure 3:**
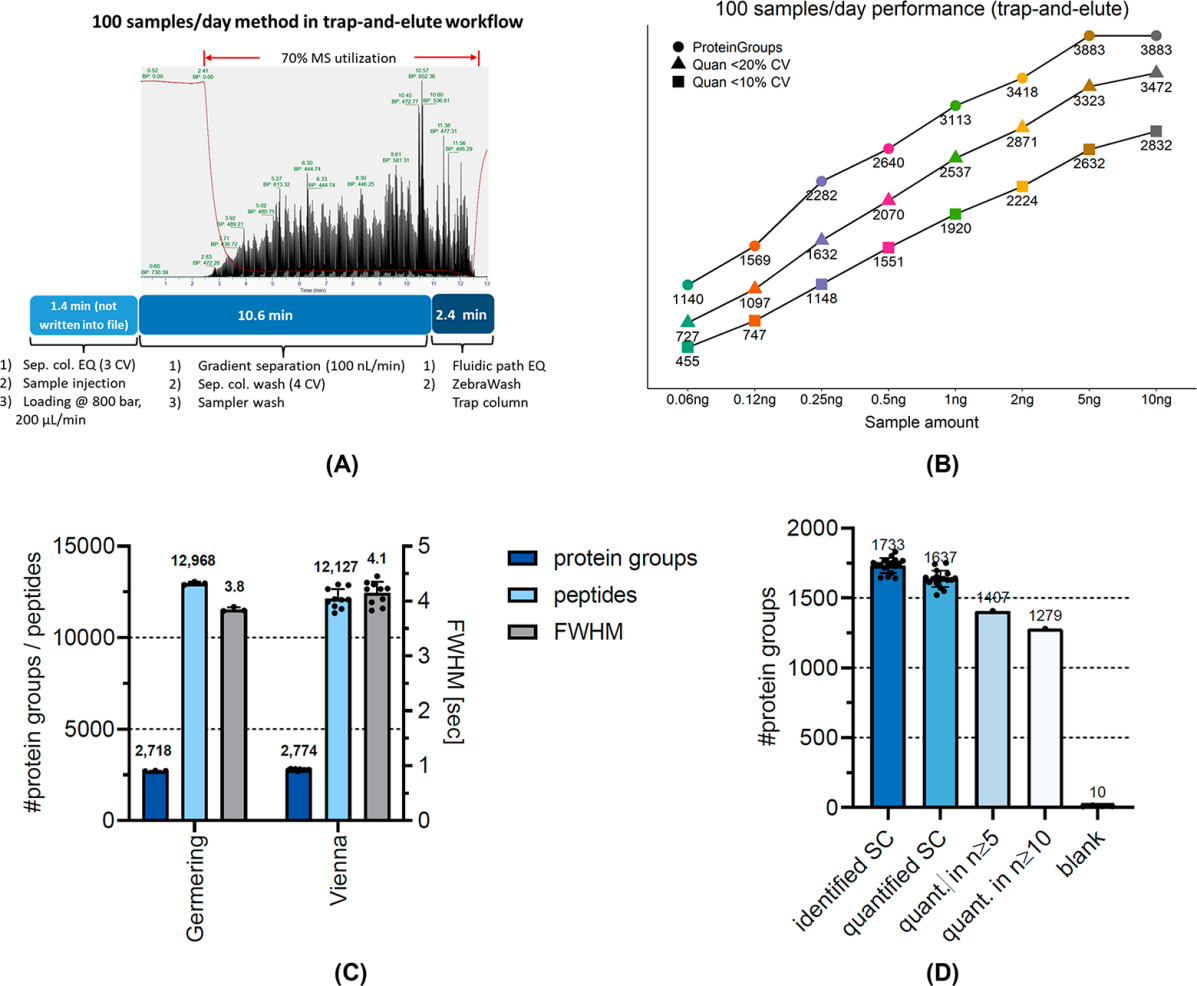
High-performance SCP analysis with 100 samples/day throughput in the trap-and-elute workflow. (A) A 14.4-min method in trap-and-elute workflow permits 100 samples/day throughput at 100 nL/min with the pump pressure trace presented by the red trend line. (B) Number of protein groups identified as a function of the sample loading amount in WW-DIA. (C) Reproducible interlab (Germering, *n* = 3; Vienna, *n* = 10) performance verifies the high sensitivity of the method in HeLa samples. (D) Achieving *>*1,700 protein groups identification from single HeLa cells (*n* = 17, one combined search in Spectronaut 17) with neglectable carryover.

To accelerate sample loading and eliminate the potential negative impact of interferants and detergent on electrospray ionization, we employed a trap column operated in a backward flush mode to maintain peak shape, successfully decreasing the method cycle time to 14.4 min (100 samples/day) for a 10 min gradient at 100 nL/min with approximately 70% MS utilization (Figure 3A). Using the optimized MS parameters from the direct injection workflow, we observed ca. 14–17% lower protein identifications in the trap-and-elute workflow (Figure 2B and 3B). Nevertheless, with the performance of over 2,200 identified protein groups in 250 pg, > 1,100 protein groups were still identified from as low as 60 pg samples (Figure 3B). Consequently, this configuration shows a similar sample throughput and MS utilization to a previously published dual-trap fluidic configuration but at a 5 times lower flow rate using a 1.5 × narrower I.D. column, achieving higher ionization efficiency and sensitivity.^18^ Ultimately, this method reproducibly identified > 2,700 protein groups from 250 pg diluted HeLa samples with WW-DIA (Figure 3C) in two laboratories, despite different operators, columns, emitters, LC-MS, etc. Lastly, the method provided >1,700 protein group identifications (>1,600 quantified) in individual HeLa cells with a library-free DIA approach (Figure 3D) in 100 cells/day (CPD) throughput. Notably, it outperformed our benchmarked configuration with another DIA acquisition method^7,19^ (Supplemental Figure 7) in three times higher sample throughput.

## Conclusion

We established a high-performance configuration for sample-limited proteomics and validated it with single-cell samples after systematically evaluating the impact of sample amount, gradient length, MS2 IT, and database search algorithms. The method utilizes a 50 μm I.D. separation column at 100 nL/min to achieve high-sensitivity results where ca. 1,700 protein groups are identified from single HeLa cells at a throughput of 100 samples/day, which outperformed our previously published results from a 32 CPD LC-MS method with the same sample preparation workflow. Overall, the above workflow provides a platform for high-sensitivity analysis of limited samples, e.g., SCP, for investigating cellular heterogeneity in clinically and biologically relevant cellular populations.

## Data Availability

All raw and result files are available for download at MassIVE^15^ using the identifier MSV000092414.

## Supporting Information Available

Method: sample preparation, liquid chromatography parameters, MS parameters, and data analysis; Supplemental Figure 1: Five optimized direct injection LC-MS methods balancing sensitivity and throughput for different proteome coverage needs; Supplemental Figure 2: The direct injection DDA methods feature low retention time variations and narrow peak widths for all different loads and gradient lengths; Supplemental Figure 3: High quantitation accuracy can be observed for the LC-MS DDA methods; Supplemental Figure 4: Improved protein group identification by CHIMERYS algorithm; Supplemental Figure 5: Protein dynamic range spanning 4 orders of magnitude for 250 pg samples using a 10-min LC gradient; Supplemental Figure 6: Direct injection workflow performance using a 20-min LC gradient; Supplemental Figure 7: LFQ-DIA analysis at 32 SPD throughput for 250 pg HeLa standard in a trap-and-elute workflow using benchmarked configuration with wishDIA acquisition method (PDF)
