# Supplementary material for: A High-Sensitivity Low-Nanoflow LC-MS Configuration for High-Throughput Sample-Limited Proteomics
Source: Anal Chem. 2023 Dec 13;95(51):18673–8. doi: 10.1021/acs.analchem.3c03058 (PMC10753523; doi:10.1021/acs.analchem.3c03058)
Supplement: Supplementary file 1 — ac3c03058_si_001.pdf [file ac3c03058_si_001.pdf]

Supporting Information

A high-sensitivity low nano-flow chromatography configuration for high-throughput MS-based sample-limited proteomics

Runsheng Zheng[1, *, #], Manuel Matzinger[2, #], Rupert L. Mayer[2], Alec Valenta[1], Xuefei Sun[3], Karl Mechtler[2, 4, 5]

[1]Thermo Fisher Scientific, Germering, Germany

[2]IMP–Institute of Molecular Pathology, Campus-Vienna-Biocenter 1, A-1030 Vienna, Austria

[3]Thermo Fisher Scientific, Sunnyvale, USA

[4]IMBA–Institute of Molecular Biotechnology of the Austrian Academy of Sciences, Dr. Bohr Gasse 3, A-1030 Vienna, Austria

[5]Gregor Mendel Institute of Molecular Plant Biology of the Austrian Academy of Sciences, Dr. Bohr Gasse 3, A-1030 Vienna, Austria

#Contribution equally

*Corresponding author: runsheng.zheng@thermofisher.com

Table of content

Method: sample preparation, liquid chromatography parameters, MS parameters, and data analysis

Supplemental Figure 1: Five optimized direct injection LC-MS methods balancing sensitivity and throughput for different proteome coverage needs

Supplemental Figure 2: The direct injection DDA methods feature low retention time variations (A) and narrow peak widths (B) for all different loads and gradient lengths

Supplemental Figure 3: High quantitation accuracy can be observed for the LC-MS DDA methods

Supplemental Figure 4: Improved protein group identification by CHIMERYS algorithm

Supplemental Figure 5: Protein dynamic range spanning 4 orders of magnitude for 250 pg samples using a 10-min LC gradient

Supplemental Figure 6: Direct injection workflow performance using a 20-min LC gradient

Supplemental Figure 7: LFQ-DIA analysis at 32 SPD throughput for 250 pg HeLa standard in a trap-and-elute workflow using benchmarked configuration with wishDIA acquisition method

Method

Sample preparation

Thermo Scientific™ Pierce™ HeLa Digest (PN: A47996, 10 µg/vial) was reconstituted by adding 100 µL of 10% ACN (v/v) with 0.1% formic acid (FA) in water and sonicated for 5 min. Subsequently, a 10 µL sample was added to 990 µL water with 0.1% FA or 0.1% trifluoroacetic acid (TFA) for direct injection or trap-and-elute applications, respectively, in a 1 mL high recovery vial (PN: 6PSV9-V1) with a talcum-free cap (PN: 6PSC9STB1), followed by a 20 - 30s vertexing and 5 min sonication to get a final concentration of 1 ng/µL for LCMS analysis. The sample amount was varied by injecting different volumes from 0.06 - 10 µL of HeLa digest onto columns.

Single-cell samples from HeLa cell lines were prepared in a 384-well plate (PN: 60180-P340) [1]. In brief, a cell suspension of 200 HeLa cells/µL in PBS was used to isolate individual cells of 18-25 µm diameter with the CellenONE® (Cellenion) into wells of a 384-well plate prefilled with 1µL of master mix containing 0.2% DDM (PN: D4641-500MG, Sigma Aldrich, Germany), 100mM TEAB (PN: 17902-500ML, Fluka Analytical, Switzerland), 3 ng/µL trypsin (Trypsin Gold, V5280, Promega, USA), 0.01 % enhancer (ProteaseMAX™, V2071, Promega, USA). Lysis and digestion were performed in parallel by incubating the plate for 30 min at 50°C. After that, another 0.5 µL 3ng/µL trypsin was added, followed by an additional 1.5 h incubation at 50°C. After that, the total sample volume was brought to 3.5 µL by adding 0.1% TFA with 1% DMSO to each well, respectively. Plates were covered by a silicon mat (PN: 60180-M150) and placed directly into the autosampler of the LC-MS device to inject 100% of every single cell sample.

Liquid chromatography parameters

The high-sensitivity, low-nanoflow chromatography configuration comprises a Thermo Scientific™ Vanquish™ Neo UHPLC system (PN: VN-S10-A-01) and a Thermo Scientific™ Acclaim™ PepMap™ 100 C_{18} 50 µm I.D. × 15 cm column (PN: 164943) with a separation flow rate of 100 nL/min. The column inlet is

connected to the sampler valve with a Thermo Scientific™ nanoViper™ capillary (10 µm x 350 mm (PN: 6250.5135) and 20 µm x 550 mm (PN: 6250.5260) for direct injection and trap-and-elute workflows, respectively) via a low-dispersion Y-piece (PN:6250.1009) and a nanoViper blind nut (PN: 6040.2303, to block the third port). The column outlet is connected to a 10 µm I.D. x 5 cm emitter (Fossillion technology, LOTUS) via a PTFE sleeve (PN: 160489) or a MicroTight unit (P771) with two sleeves (SC603 and SC903 for emitter inlet and column outlet, respectively). A Sonation holder (PN: 004.800.01) insulated the Y-piece where the voltage was applied. The column was heated to 50 ^{o}C via a Sonation source (PN: PRSO-V2-ES72) with or without an inlay (PN: PRSO-V2-IZDV-72) for the MicroTight unit or PTFE sleeve, respectively. Mobile phase A and weak wash liquid were water with 0.1% FA (P/N LS118−500), and mobile phase B and strong wash liquid were 80% acetonitrile with 0.1% FA (P/N LS122500). All solvents were from Thermo Fisher Scientific. The autosampler temperature was 7 °C.

In the direct injection workflow, samples were loaded at 1500 bar with 2 uL loading volume using pressure control mode and separated at 100 nL/min, followed by 0.1 column volumes for column equilibration with the Fast Equilibration option off. Five LC methods were developed that enable 72, 60, 48, 36, and 24 samples daily with 10, 14, 20, 30, and 50-min separation gradients, respectively.

In the trap-and-elute workflow, a 14.4-min method with a 10-min gradient was developed to enable 100 runs/day. A 300 µm x 5 mm PepMap trap cartridge was installed on the sampler valve. Samples were loaded at 800 bar with 5 uL loading volume using pressure control mode, and at the end of the gradient separation, the trap cartridge was automatically switched offline for a 4-cycle ZebraWash and re-equilibration with a default value (i.e., twice the ZebraWash volume). The separation was run at 100 nL/min with a flow ramp at the beginning and end of the separation (see gradient table below), followed by 0 column volume for column equilibration with the Fast Equilibration option off.

The gradient details are given below.

72 samples/day (direct injection)

Time (min)	Duration (min)	Flow rate (µL/min)	%B
Gradient Separation Phase			
0	0	0.5	1
0.5	0.5	0.1	1
1	0.5	0.1	8
8	7	0.1	20
10	2	0.1	35
Column Wash Phase			
10.1	0.1	0.1	99
14	3.9	0.1	99

60 samples/day (direct injection)

Time (min)	Duration (min)	Flow rate (µL/min)	%B
Gradient Separation Phase			
0	0	0.5	1
0.5	0.5	0.1	1
1	0.5	0.1	8
11.2	10.2	0.1	20
14	2.8	0.1	35
Column Wash Phase			
14.1	0.1	0.1	99
18	3.9	0.1	99

48 samples/day (direct injection)

Time (min)	Duration (min)	Flow rate (µL/min)	%B
Gradient Separation Phase			
0	0	0.5	1
0.5	0.5	0.1	1
1	0.5	0.1	8
16	15	0.1	20
20	4	0.1	35
Column Wash Phase			
20.1	0.1	0.1	99
24	3.9	0.1	99

36 samples/day (direct injection)

Time (min)	Duration (min)	Flow rate (µL/min)	%B
Gradient Separation Phase			
0	0	0.5	1
0.5	0.5	0.1	1
1	0.5	0.1	8
24	23	0.1	20
30	6	0.1	35
Column Wash Phase			
30.1	0.1	0.1	99
34	3.9	0.1	99

24 samples/day (direct injection)

Time (min)	Duration (min)	Flow rate (µL/min)	%B
Gradient Separation Phase			
0	0	0.5	1
0.5	0.5	0.1	1
1	0.5	0.1	8
40	39	0.1	20
50	10	0.1	35
Column Wash Phase			
50.1	0.1	0.1	99
54	3.9	0.1	99

100 samples/day (trap-and-elute)

Time (min)	Duration (min)	Flow rate (µL/min)	%B
Gradient Separation Phase			
0	0	0.5	2
1.4	1.4	0.5	13
2.4	1	0.5	25
2.5	0.1	0.1	99
4.5	2	0.1	99
Column Wash Phase			
10.5	6	0.1	99
10.6	0.1	0.1	1
12.4	1.8	0.1	1
12.5	0.1	0.5	1
13	0.5	0.5	1

MS parameters

The data was acquired on a Thermo Scientific™ Orbitrap Exploris™ 480 mass spectrometer with a Thermo Scientific™ FAIMS Pro™ interface. Three data acquisition strategies, DDA, WWA, and WW-DIA, were employed to evaluate method sensitivity and performance. In positive mode, the spray voltage was set at 1800-2200 V, and the ion transfer tube temperature was set at 275 °C. RF lens was set at 45%. FAIMS compensation voltage was set to −50 V with a carrier gas of 3.5 L/min.

For DDA experiments, the Orbitrap Exploris MS was operated at a full scan with an m/z range of 375−1200, Orbitrap resolution of 120,000, normalized target value 300%, and maximum injection time set to Auto. The intensity threshold for the precursor was set to 5×10^3. MS/MS spectra starting from 120 m/z were acquired with 10 dependent scans, where the precursors were isolated in a window of 2.0 Da and subsequently fragmented with HCD using an NCE (normalized collision energy) of 26%. Orbitrap resolution was set to 60,000. The normalized AGC target was 50%, and the maximum injection time was 118 ms.

For WWA experiments, all the settings are identical to the DDA experiment, except the isolation window varies from 2-12 m/z.

For DIA experiments, the Orbitrap Exploris MS was operated at a full scan with an m/z range of 400−800, Orbitrap resolution of 120,000, a normalized target value of 300%, and a maximum injection time set to Auto. For evaluation, the fixed precursor isolation window was set at 20, 30, 40, 50, 60, 80, and 100 m/z, respectively, over the gradient with the window placement optimization function enabled. After that, the fixed isolation window of 40 m/z was selected for applications. The subsequent fragmentation was performed with an HCD collision energy of 28%, and spectra were acquired at a minimum of 120 m/z at an Orbitrap resolution of 60,000 in centroid mode. The normalized AGC target was 1000%, and the maximum injection time was 118 ms.

Data analysis

Raw files from replicates with identical conditions, e.g., LC gradient, sample amount, and MS parameters, were searched together to enable reasonable parameter evaluation in this study.

The DDA dataset was processed with the Thermo Scientific™ Proteome Discoverer™ 2.5 software using a 2-step SEQUEST™HT search algorithm and INFERYS™ rescoring node (available as template). Data were searched against the human reference database from SwissProt (including isoforms). Trypsin was specified as the proteolytic enzyme with up to 2 missed cleavages allowed, and Carbamidomethyl as the static modification. The first step of SEQUEST™ HT search enabled Oxidation (M) as the dynamic modification, while the second step search included 6 dynamic modifications: Deamidated (N,Q), Oxidation (M), Gln→pyro-Glu (Q), Acetyl (N-terminus), Met-loss + acetyl (M), and Met-loss (M). The peptide peak widths were calculated with the apQuant node [2].

With the default processing workflow template, the chimeric spectra in the DDA and WWA datasets were searched with the CHIMERYS™ algorithm in the Proteome Discoverer™ 3.0 with Carbamidomethyl (C) as a fixed modification and Oxidation (M) as a variable modification. All DDA results above were reported without match-between-run (MBR).

DIA files were submitted to Spectronaut 17 (version 17.2.230208.55965) for peptide and protein identification and quantification in directDIA™ analysis without a library using the same human reference database in the DDA searches. Peptide fixed modification was set to Carbamidomethyl for all samples except the single cells, while acetyl (Protein N-term) and oxidation (M) were applied to all as variable modifications. Only the identification by MS/MS spectrum was reported, and the false discovery rates (FDR) were all set below 1% at both the peptide and the protein levels.

Further data analysis and plotting were performed with R script [3] and GraphPad Prism.

Supplemental figures

Sample Throughput/Day	Cycle Time (min)	Gradient Length (min)	Elution Window (min)	Sample Injection & Loading (min)*	MS utilization (%)
72	20	10	11	5.4	55
60	24	14	14	5.4	62.5
48	30	20	21	5.4	70
36	40	30	31	5.4	77.5
24	60	50	51	5.4	85

* 1 µL sample injection + 2 µL loading

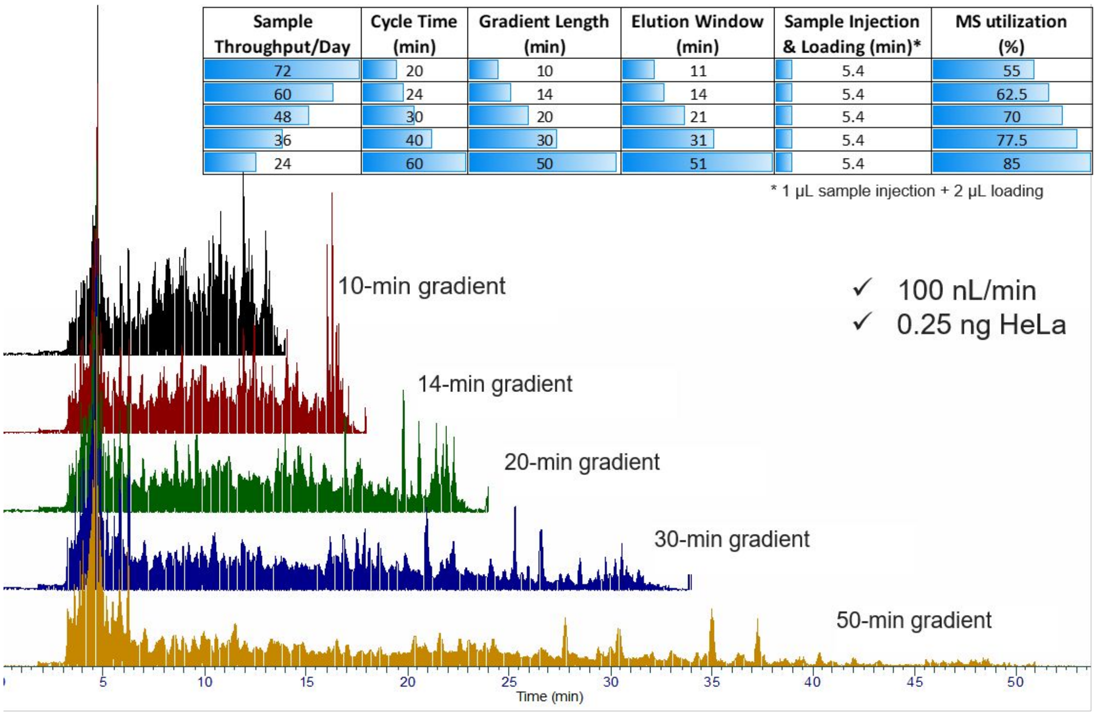

Supplemental Figure 1. Five optimized direct injection LC-MS methods balancing sensitivity and throughput for different proteome coverage needs

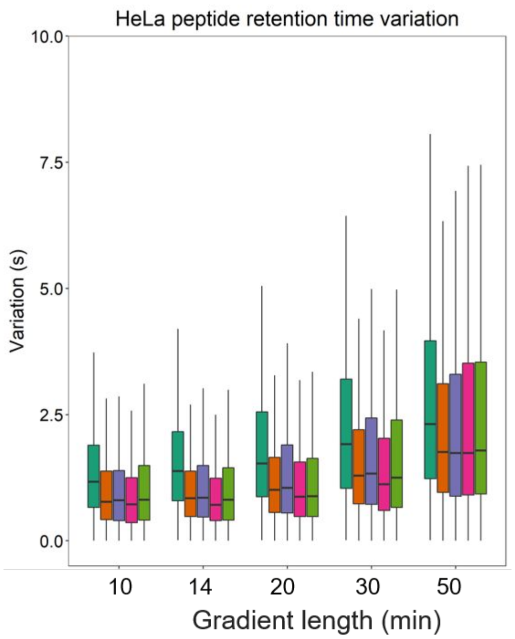

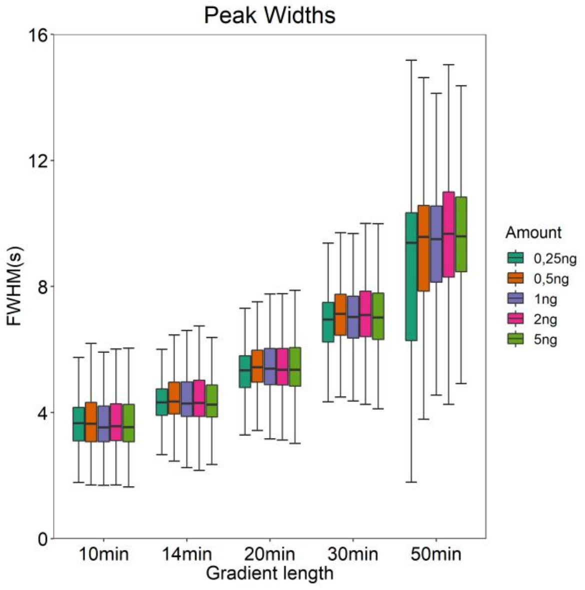

(B)

(A)

Supplemental Figure 2. The direct injection DDA methods feature low retention time variations (A) and narrow peak widths (B) for all different loads and gradient lengths

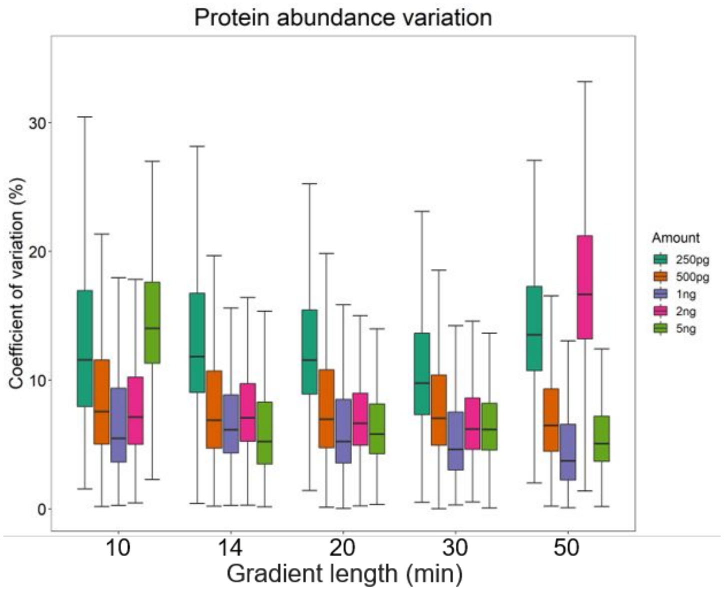

Supplemental Figure 3. High quantitation accuracy can be observed for the LC-MS DDA methods

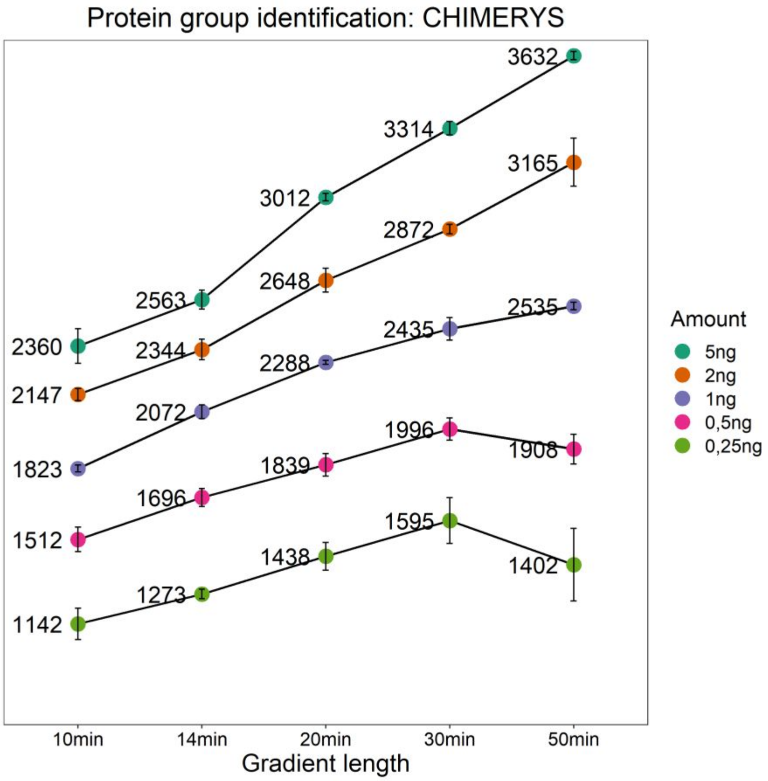

Supplemental Figure 4. Improved protein group identification by CHIMERYS algorithm

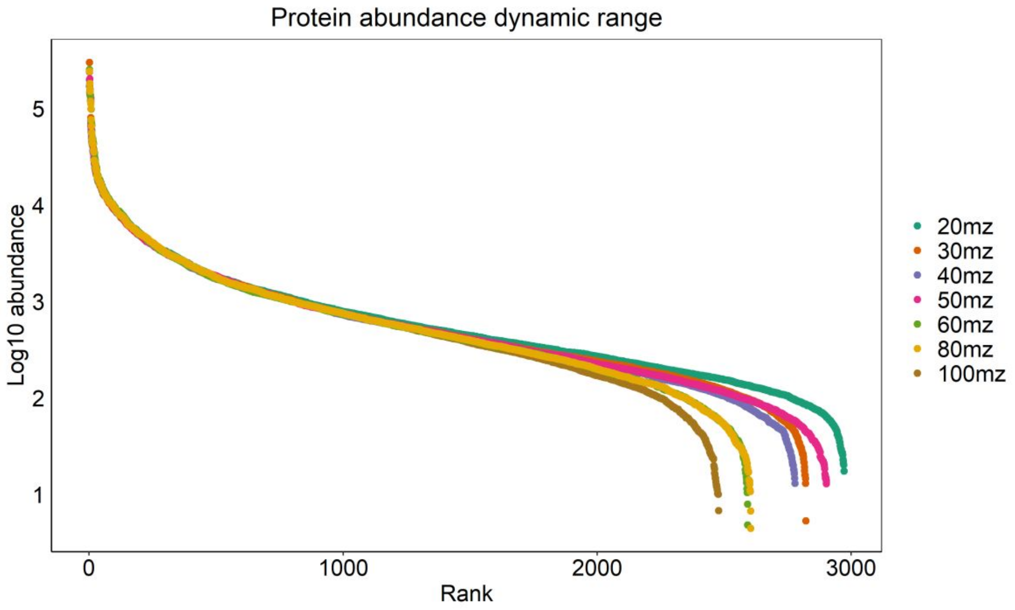

Supplemental Figure 5. Protein dynamic range spanning 4 orders of magnitude for 250 pg samples using a 10-min LC gradient

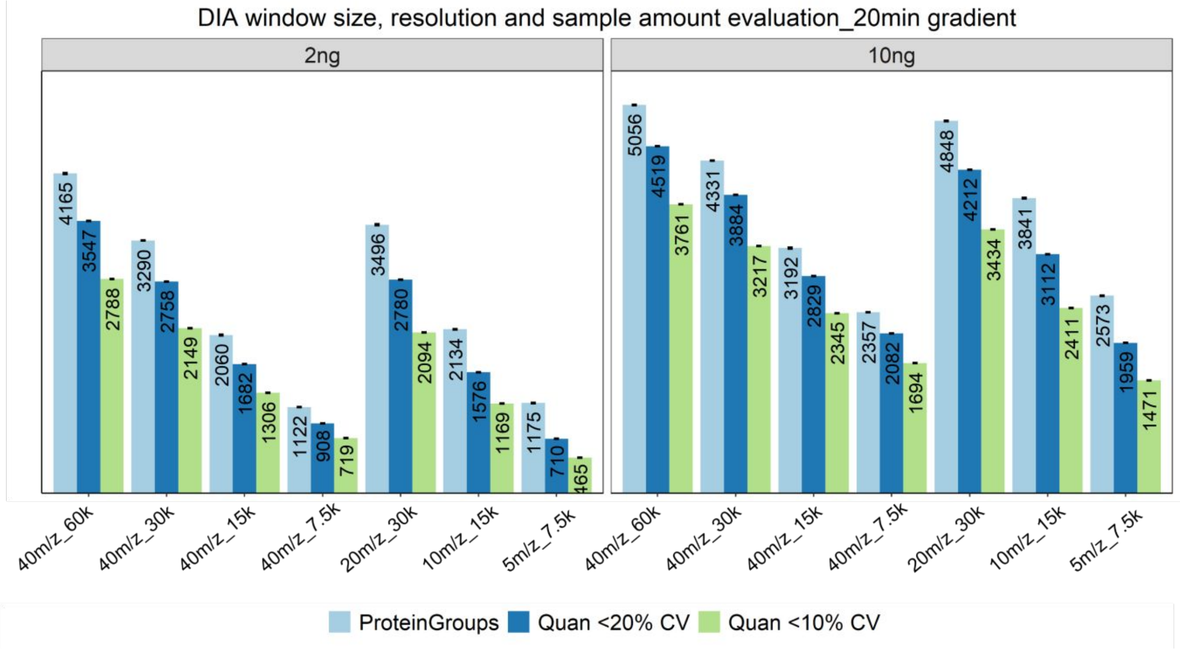

Supplemental Figure 6. Direct injection workflow performance using a 20-min LC gradient

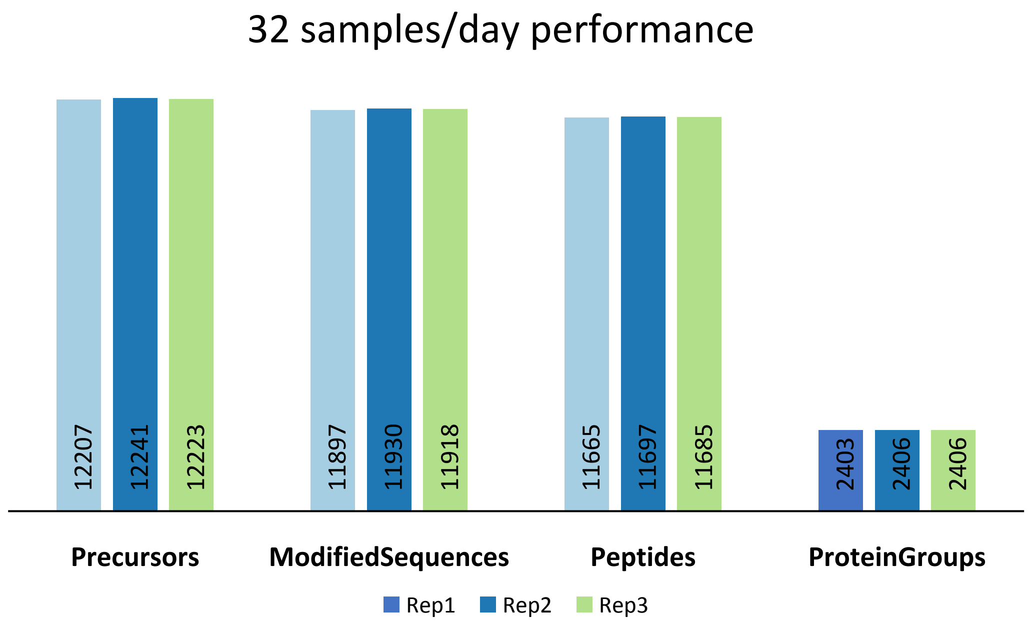

Supplemental Figure 7. LFQ-DIA analysis at 32 SPD throughput for 250 pg HeLa standard in a trap-and-elute workflow using a benchmarked configuration with wishDIA acquisition method

Reference

(1) Matzinger, M.; Müller, E.; Dürnberger, G.; Pichler, P.; Mechtler, K. Robust and Easy-to-Use One-Pot Workflow for Label-Free Single-Cell Proteomics. *Anal. Chem.* 2023, 95 (5), 4435-4445. https://doi.org/10.1021/acs.analchem.2c05022.

(2) Doblmann, J.; Dusberger, F.; Imre, R.; Hudecz, O.; Stanek, F.; Mechtler, K.; Dürnberger, G. apQuant: Accurate Label-Free Quantification by Quality Filtering. J Proteome Res. 2019, 18(1):535–541. https://10.1021/acs.jproteome.8b00113.

(3) R Core Team (2020) R: A Language and Environment for Statistical Computing. R Foundation for Statistical Computing, Vienna, Austria. Https://Www.r-Project.Org/.
